# Elderly female ultra-marathoners reduced the gap to male ultra-marathoners in Swiss running races

**DOI:** 10.1038/s41598-023-39690-6

**Published:** 2023-08-02

**Authors:** Beat Knechtle, Anja Witthöft, David Valero, Mabliny Thuany, Pantelis T. Nikolaidis, Volker Scheer, Pedro Forte, Katja Weiss

**Affiliations:** 1 Medbase St. Gallen am Vadianplatz, Vadianstrasse 26, 9001 St. Gallen, Switzerland; 2 Institute of Primary Care, University of Zurich, Zurich, Switzerland; 3 Kinderspital St. Gallen, St. Gallen, Switzerland; 4 Ultra Sports Science Foundation, Pierre-Benite, France; 5 Faculty of Sports, University of Porto, Porto, Portugal; 6 School of Health and Caring Sciences, University of West Attica, Athens, Greece; 7 CI-ISCE, Higher Institute of Educational Sciences of the Douro, Penafiel, Portugal; 8 Instituto Politécnico de Bragança, Bragança, Portugal; 9 Research Center in Sports, Health and Human Development, Covilhã, Portugal

**Keywords:** Environmental sciences, Environmental social sciences

## Abstract

Recent studies showed that female runners reduced the performance gap to male runners in endurance running with increasing age and race distance. However, the investigated samples were generally small. To investigate this further, the present study examined sex differences by age across various race distances (5, 10 km, half-marathon, marathon, and ultra-marathon) using a large dataset of over 1,100,000 race records from Switzerland over two decades (1999–2019). The study explored performance and participation disparities between male and female runners by employing diverse methods, such as descriptive statistics, histograms, scatter and line plots, correlations, and a predictive machine learning model. The results showed that female runners were more prevalent in shorter races (5, 10 km, half-marathon) and outnumbered male runners in 5 km races. However, as the race distance increased, the male-to-female ratio declined. Notably, the performance gap between sexes reduced with age until 70 years, after which it varied depending on the race distance. Among participants over 75 years old, ultra-marathon running exhibited the smallest sex difference in performance. Elderly female ultra-marathoners (75 years and older) displayed a performance difference of less than 4% compared to male ultra-marathoners, which may be attributed to the presence of highly selected outstanding female performers.

## Introduction

Running is high popular^1^ and is organized in different event formats such as track running^2,3^, road running^4^ and trail running (off-road running)^5^. Road-based running races are held over different distances, such as 5 km^6^, 10 km^4^, half-marathon^7^, marathon^8^ and ultra-marathon^9^. Recently, the interest in sports science to study female athletes profiles has grown (i.e., physiology, professionalism, and contextual factors that affect performance)^10,11^, with the aspect of sex difference in running garnering high-interest^12–17^. Twenty years ago, it was assumed that longer running distances were associated with higher sex differences. This might have been confounded by the reduced number of female runners in longer running distances^15^. It was also assumed that a sex difference of ~ 11–12% would be unchanged independent of the distance^15^ and would not change over years^17,18^. Today, the sex difference is still higher in longer running distances compared to shorter distances^16^, and the sex difference of different sports disciplines remained stable at ~ 10%^12^. It highlighted the need for researching the sex differences in different running distances.

Recent studies showed that elderly female ultra-marathoners reduced the gap to male ultra-marathoners of the same age^13,14^. Age seems to be of higher importance than the length of a race. It has been shown that female runners reduced the gap to male runners with increasing age, not with the increasing length of a race^14^. It is important to understand the contextual and environmental factors that may explain sex differences in running competitions. Modality popularity is dependent on the number of participants and competitive level. Therefore, females reducing the performance gap to male runners may improve participation in the sports modality. Upon that, it is important to understand the performance differences between sex (i.e., running speed), the ratio of participants between sexes and the evolution of the participant number over time to explain the sex differences in running. This first step may allow forecasting strategies to reduce sex differences in running.

Studies investigating the sex difference in endurance running performance analyzed rather small samples and/or single distances^19^. The present study investigated the sex difference in performance in running races of 5, 10 km, half-marathon, marathon, and ultra-marathon with race records of two decades of a single country with a sample size of more than one million race records. Based upon recent findings regarding the reduction of the sex difference in longer running distances and older age groups, we hypothesized to confirm recent findings.

## Methods

### Ethical approval

This study was approved by the Institutional Review Board of Kanton St. Gallen, Switzerland, with a waiver of the requirement for informed consent of the participants as the study involved the analysis of publicly available data (EKSG 01/06/2010). The study was conducted in accordance with recognized ethical standards according to the Declaration of Helsinki adopted in 1964 and revised in 2013.

### Data set

Athletes’ data from all 5, 10 km, half-marathon, marathon and ultra-marathon races held in Switzerland between 1999 and 2019 were collected from different sources such as “swiss-running” (www.swiss-running.ch), “runme” (www.runme.ch/de/laufkalender/schweiz), “datasport” (www.datasport.com/de) and “DUV” (https://statistik.d-u-v.org/calendar.php). For all race distances, road-based races and trail runs were included. For ultra-marathons, any running races longer than the official marathon distance of 42.195 km and longer than 6 h were combined^20^.

### Data preparation

For each race, data from successful participants, including name and surname, sex, age, year of birth, distance and race time, event name and terrain type, were obtained from the websites and recorded in an EXCEL file. The average running speed (km/h) was calculated from the race distance and time. While the continuous age variable was available, race records were also classified (and later aggregated) by age group according to the official 5-year age group intervals. Records from runners younger than 18 were discarded. The junior category (18 years) includes only runners aged 18 and 19, while runners older than 75 were considered 75+ years. Race records were also aggregated by year for male and female runners separately. The male-to-female ratio was calculated by dividing the number of male records by the number of female records each year.

### Statistical analysis

Data normality was assessed by plotting histograms of the race speed in each race distance and for each sex. Descriptive statistics is then presented through the mean and standard deviation. For each race distance and age group, the male-to-female ratio was calculated by dividing the number of male records by the number of female records in each age group. The percentage difference in average speed was calculated as 100 * (male speed − female speed)/male speed. Pearson correlations were calculated between the male-to-female ratio, the percentage difference of speed and the age, where the male-to-female ratio and percentage of speed difference were calculated for each year of age (18 through to 100). Statistical significance was assessed through the calculation of p-values, where the threshold set at *p* < 0.05. A machine learning (ML) predictive model was built and evaluated through the R^2^ and MAE metrics, with further analysis through SHAP values and feature importance/interaction analysis.

All data processing, analysis and visualization were done in a Google Colab notebook using Python and statistical/machine learning free packages such as numpy, pandas, matplotlib, seaborn, statsmodels, scipy.stats, sklearn, catboost, shap.

## Results

A total of 1,149,182 race records from 419,042 runners competing in 243 race events were considered. Table 1 presents the number of runners by distance and sex. Female runners were more numerous than male runners in the 5-km run. The male-to-female ratio increased with increasing race distance.

**Table 1:** Number of male and female runners by race distance and male-to-female ratio.

Race distance	Male	Female	Total	Male-to-female ratio
5 km	44,866	56,954	101,820	0.78
10 km	204,648	125,469	330,117	1.63
Half-marathon	268,486	117,275	385,761	2.28
Marathon	187,778	43,777	231,555	4.28
Ultra-marathon	83,040	16,889	99,929	4.91

**Figure 1:**
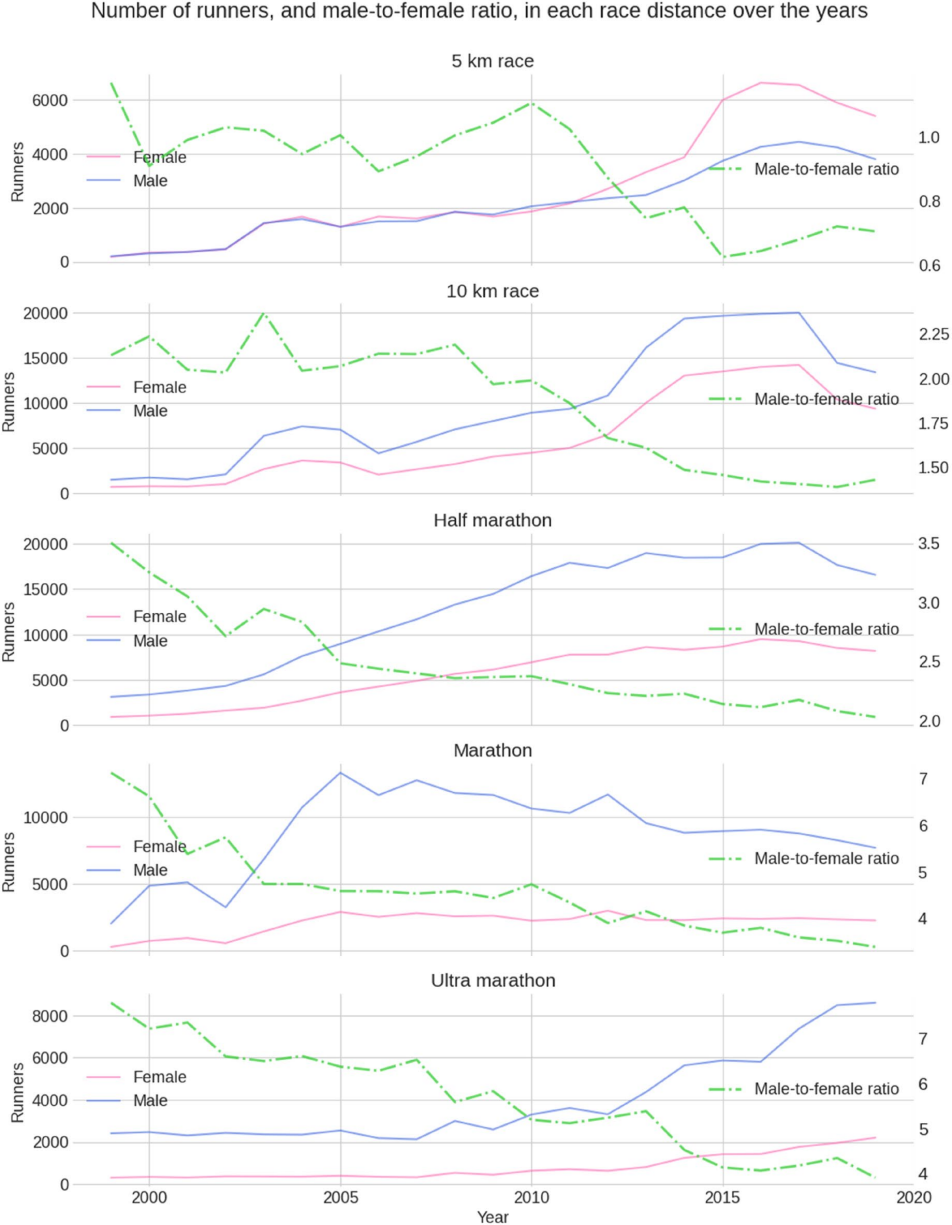
The trend in the number of runners and male-to-female ratio over the years by distance and sex.

Figure 1 summarizes the trend in the number of runners over the years by distance and sex, along with the male-to-female ratio. The male-to-female ratio generally decreased in all race distances over the years, caused by a more progressive increase in the number of female runners (Fig. 1, pink lines) compared to the number of male runners (Fig. 1, blue lines). The number of male marathoners in Swiss races has steadily decreased since 2005.

**Figure 2:**
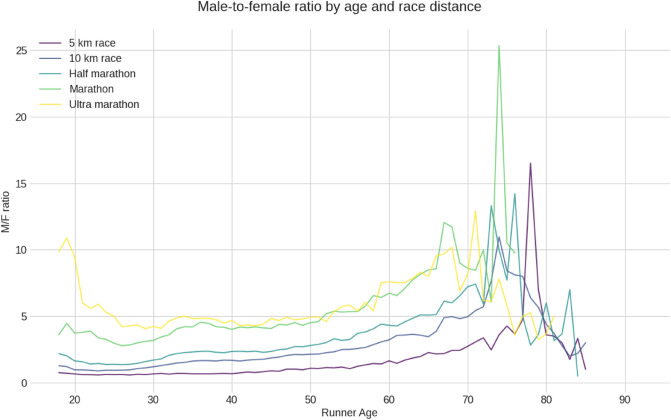
The male-to-female ratio across age groups for all race distances.

Figure 2 shows the male-to-female ratio by age group and race distance. There is a general growing trend in the ratio from age 25 years until age group 70 years. Thereafter, the ratio increased further in 5 km and marathon, remained flat in 10 km, but decreased in half-marathon and ultra-marathon.

**Figure 3:**
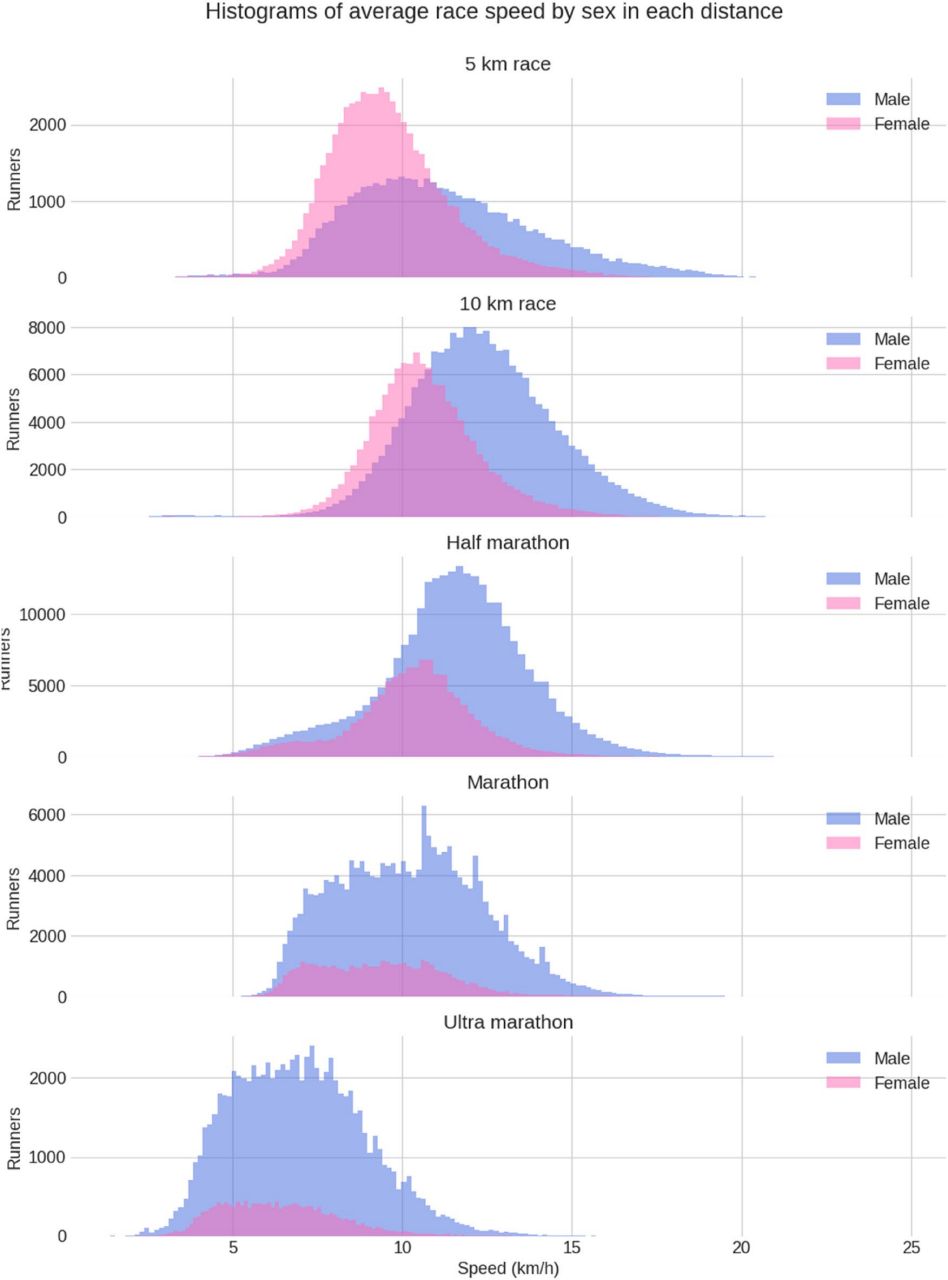
Histograms of average running speed by distance and sex.

Figure 3 presents the histograms of the average running speed by distance and sex. In general, male runners were running faster than female runners in all race distances. The fastest average running speed was achieved at 10 km for both female and male runners. According to descriptive statistics, female runners participated more in the shorter distances and less in marathons and ultra-marathons, but the performance by sex did not differ so much in the longer distances.

**Figure 4:**
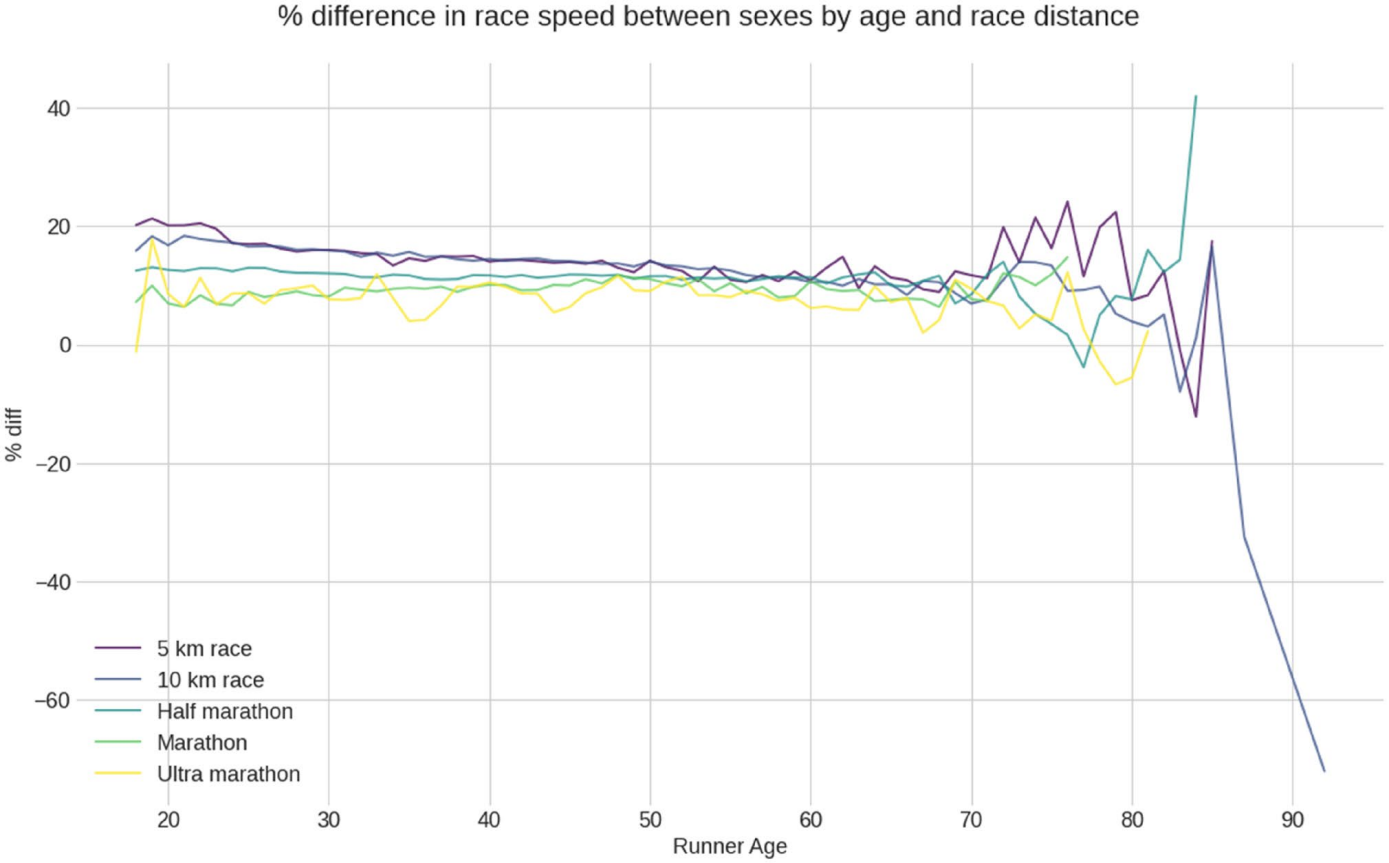
% speed difference by sex for all race distances.

Figure 4 presents the sex difference in percent difference by age for the five-race distances. According to descriptive statistics, there is a decreasing trend from the age 20 years to age 70 years in the 5, 10 km, and half marathon distances. This decreasing trend starts at 45 years in marathon distances and at age 50 years in ultra-marathons. In 5 km and marathon distances, the sex difference increases after age 65 years, while in 10 km, half-marathon and ultra-marathon, the sex difference continues decreasing beyond this age.

Table 2 presents the associations between the male-to-female ratio versus the % difference in running speed, between the male-to-female ratio and age group, and between the % difference in running speed and age group.

**Table 2:** Correlations between the male-to-female ratio, % difference in running speed, and age (r = correlation coefficient, *p* = *p* value).

	5 km		10 km		Half-Marathon		Marathon		Ultra-marathon	
	r	*p*	r	*p*	r	*p*	r	*p*	r	*p*
M/W ratio vs % running speed difference	0.04	0.73	− 0.01	0.92	− 0.45	0.0001	0.28	0.03	0.025	0.84
M/W ratio vs age	0.57	2.97e−07	0.66	6.3e−10	0.67	6.9e−10	0.73	2.8e−11	0.18	0.16
% running speed difference vs age	− 0.43	0.0002	− 0.55	7.1e−07	− 0.11	0.35	0.39	0.001	−0.42	0.0004

According to descriptive statistics, the male-to-female ratio increased with age, with some exceptions in the youngest and the oldest age. Furthermore, there were always more male than female runners (except for 5 km), and males were always faster than females, where the differences in running speed declined with increasing age, except in the marathon distance.

In 5 km, we found a significant and positive association (r = 0.57, *p* < 0.05) between the male-to-female ratio and the age and a significant and negative association (r = − 0.43, *p* < 0.05) between the difference in running speed and age. In 10 km, similar significant correlations can be observed, with slightly higher values. In the half-marathon, we found a significant and negative association (r = − 0.45, *p* < 0.05) between the male-to-female ratio and the percent difference in running speed and a significant and positive correlation (r = 0.67, *p* < 0.05) between the M/W ratio and the age. In the marathon, there was a significant and positive association (r = 0.28, *p* = 0.03) between the male-to-female ratio and the percent difference in running speed and a significant and positive association (r = 0.73, *p* < 0.05) between the male-to-female ratio and the age. Last, a significant and negative association (r = − 0.42, *p* = 0.0004) between the percent difference in running speed and age was found in ultra-marathon running.

### Predictive model

To explore potential non-linear relationships between the variables of interest, an ML tree-ensemble/gradient boosting model was built and evaluated. The model uses the Cat Boost Regressor algorithm with 200 learners to predict the average race speed (km/h) from the runner´s age and sex, the distance (km) and the terrain type (flat/trail).

The model was trained with over 860 K race records or 75% of the full sample of 1,149,182, and later evaluated over the remaining 287 K records (25%), achieving predictive accuracy scores of R^2^ = 0.53, MAE = 1.32 km/h. The model features relative importance were also computed (Terrain 44%, Distance 32%, Sex 18%, Age 6%) along with the SHAP values and feature interactions.

Predictive model key indicators

**Table:** 

Sample size	1,149,182
CatBoost model	200 trees
MAE (km/h) 1.32	
R^2^ 0.53	

Features relative importances

**Table:** 

Terrain_type	44.13
Distance_km	32.06
Runner_sex	17.66
Runner_age	6.14

### SHAP summary plots

**Figure 5:**
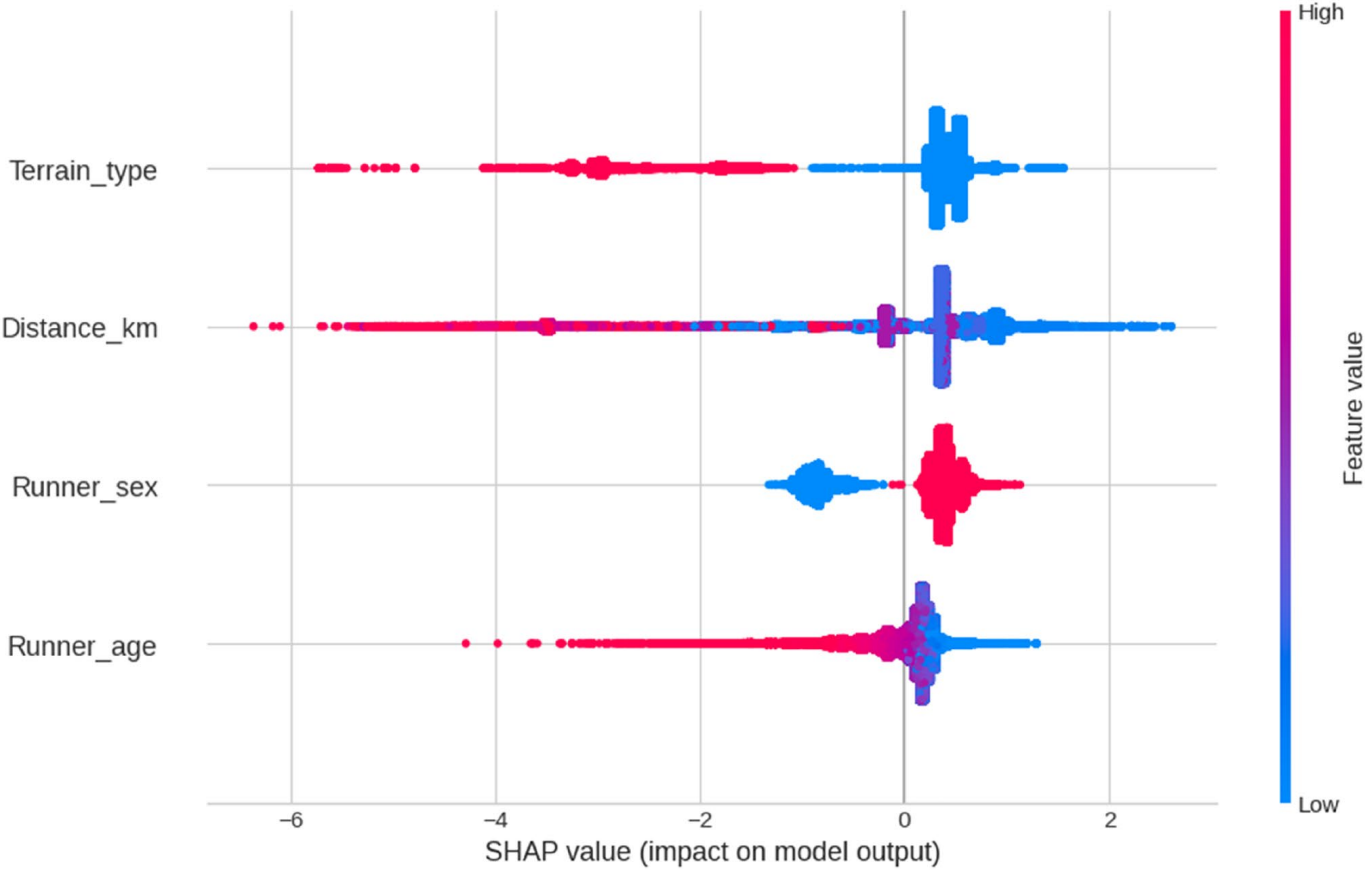
SHAP summary plots.

Figure 5 presents the SHAP summary plots of individual prediction dots relative to reference value zero. They show how the model distributes its predictions for different values of each feature.

Terrain and Distance make the broadest contributions to the model output and are rated as the model’s most important features. Flat races (Terrain_type = 0, blue dots) score on the positive side of the x-axis (higher speeds), while trail races (Terrain_type = 1, red strip of dots) get reduced speeds. A similar pattern can be observed in the distance chart. Longer distances (red dots, but also violet ones—distance is continuous) can be seen increasingly towards the left (negative side of the x-axis), whilst low values of the distance predictor accumulate in the positive side. The sex distinction is clear, with males ('1', red dots) accumulating almost completely on the right side of the chart. Finally, the age predictor, also a continuous variable, shows a similar pattern to the first two: lower ages (blue points) obtain the model's best predictions of speed, and from there the x-axis turns darker blue, purple, and red towards the left as the age increases.

### SHAP dependence plots

**Figure 6:**
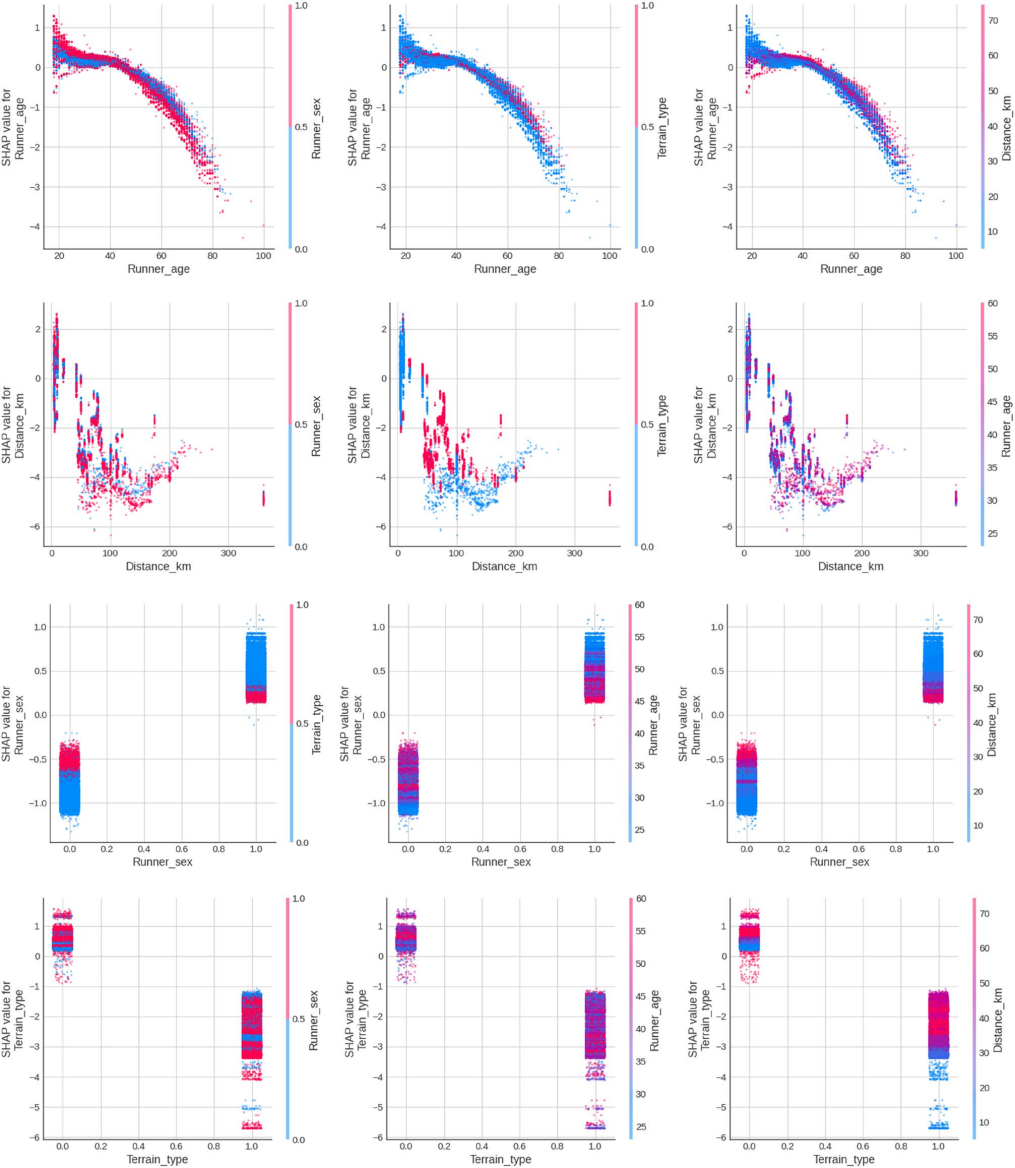
SHAP dependence plots for age of the runners, race distance, sex, and terrain (from top to down).

Figure 6 shows the SHAP dependence plots for the age of the runners, race distance, sex, and terrain. Each row represents the SHAP values for one predictor while its interactions with the other tree predictors are shown in each column. Regarding age, the first chart shows the blue dots corresponding to female, and the red dots to male, and shows an interesting trend changing gradually between 40 and 50 years. The model gives females better predictions than males from the age of 50 and up to 60 or 65 years. The second chart is largely dominated by blue (Terrain 0, flat races) and just shows the performance decline with age. The third chart has some interest, given that some red dots (long-distance races) obtain better speeds in age ranges (between 30 and 50 years and between 60 and 80 years). A possible explanation is the specialization of runners.

For the race distance predictor, the first chart shows a broad dispersion (blue and red dots overlapping) across the distance axis, indicating a small interaction of sex with the distance. An interesting chunk of blue points (Runner_sex 0, female) can be seen from the 150 km distance, indicating the model gives a significant number of higher speed predictions to female than to male runners in these long distances. The second chart shows that blue (flat run) dominates high-speed predictions for very short distances, but then red is above blue from approx. 50 km and up to 150 km. The third chart (distance/age interaction) shows equally a significant dispersion, where runners of all ages get the full range of predictions across all distances.

For sex, the first chart shows red dots (trail race predictions) closer between male and female runners, with the larger differences being between the flat races (blue points). The second chart shows that female performance (Sex 0, left bar) is more distant to male performance in the early ages (blue points) or, in other words, the model predicts the performance gap decreases with age. A similar pattern can be seen in the third chart, where blue points (performance in short-distance races) present the largest difference between male and female runners, progressively coming closer through violets and red colors (mid and old ages).

For terrain, the first chart shows that in trail races male runners obtain the lowest model predictions (red strip at the bottom of the right bar). The standard deviation of the data in the second chart is relatively high, indicating significant dispersion. The third and last chart shows that mid distances get higher speed predictions than high and short distances in both flat and trail races. This is more noticeable in trail terrain (ID 1).

## Discussion

This study investigated the sex difference in running performance in a sample size of more than one million race records with the hypothesis to confirm the recent finding of reducing the sex difference in longer running distances and older age groups. The main important findings were (i) female runners participated more in the shorter race distances and less often in marathons and ultra-marathons, (ii) female runners were more numerous than male runners in the 5-km races, (iii) the male-to-female ratio increased with increasing race distance, (iv) the male-to-female ratio decreased in all race distances over the years, (v) the number of male marathoners continuously decreased since 2005, (vi) male runners were running faster than female runners in all distances, (*vii*) the fastest average running speed was in the 10 km events, and (viii) female runners reduced the performance gap with male runners gradually from age group 20–24 years to age group 65–69 years in 5, 10 km and half-marathon, but not so much in marathon and ultra-marathon, with differences thereafter for the different race distances.

### Smallest sex difference in ultra-marathon running

The smallest sex difference was found in the longest race distance (ultra-marathon) and oldest age group (75+), supporting the hypothesis. Female runners reduced the gap to male runners in ultra-marathon by increasing the age group from 50 to 54 years. The male-to-female ratio decreased after age group 65–69 years in ultra-marathon, while it increased in marathons. The male-to-female ratio increased with age in most race distances but not in ultra-marathon. The decrease in sex difference and the increase in male-to-female ratio with increasing age was due to a higher number of faster and more competitive female runners in older age groups. This pattern was consistent across most race distances, except for an outlier in the oldest age group in marathons. The correlations between running speed difference, male-to-female ratio, and age group were similar across all race distances, with the few female runners in older age groups and ultra-marathon likely being exceptional performers.

A study of ultra-marathoners in timed and multi-day events found that peak performance age increased with longer race durations and more race finishes, indicating that successful ultra-marathoners improved with age and experience^21^. A study investigating the effect of age and years of running experience in runners aged from 20 to 80 years showed that the number of years of running had a positive effect on running economy^22^. Master ultra-marathoners improved their performance in long ultra-marathons such as ‘Badwater’ and ‘Spartathlon’^23^. It was also shown by Van den Berghe et al. that a low-loading and high-load-bearing tolerance running style could be advantageous for completing endurance running events for long-term runners^24^. A study comparing 50- and 100-mile races showed that female runners reduced the gap to male runners with increasing age, where the sex difference was smaller in 100 miles compared to 50 miles^13^. Another study investigating data from the American Master Road Running Records from 5 km to 144 h showed that female runners reduced the gap to male runners with increasing age, not with increasing length or duration of the performance^14^.

The sex difference in performance can be due to different reasons such as biological differences (e.g., muscle mass, body fat, body size, muscle strength, limb length)^25–28^, physiological differences (e.g., aerobic capacity, running economy, fatigue resistance, substrate efficiency, energetic demands)^29–31^, participation^9,32^, experience and decision making^33,34^, motivation^35^, sociocultural^36^, and psychological differences^3,37^. Elderly female runners may have advantages over elderly male runners due to their ability to store and use elastic energy more efficiently, as studies have shown^27^. Female runners have a higher proportional area of type I muscle fibers and are better at using fatty acids during prolonged exercise and preserving carbohydrates^25,38^. This may lead to a more even pacing and less fatigue than male runners^25,38^. Considering physiological differences, it has been assumed that female ultra-marathoners have better fatigue resistance compared to equally trained male ultra-marathoners who are faster in the marathon than the female ultra-marathoners^30^. Since males have a higher aerobic capacity and a higher skeletal muscle mass than females, the distance running gap will not narrow between females and males^28^. Finally, decreasing testosterone levels due to aging also compromises male physical performance^39^. However, female runners seem more prone to support fatigue, so the aging effects in performance may be lower^30^. This may also support the reduced gap in sex differences with aging.

Participation of females in ultra-marathon is an important aspect since the percentage of female runners is generally low in these races^9,40^. Lower participation of female runners compared to male runners overestimates the decline in age-related performance, especially in very old females^41^. A small sex difference in ultra-marathon running is more likely due to a low number of participants than an outstanding physiology^42^. An analysis of 20 ultra-marathons from 45 to 160 km showed that the sex difference in running was lower in the longer distances and the largest when fewer female and male runners were in a race^43^.

Another aspect is competitiveness^3,35,44–46^. It is well-known that males are more engaged in direct competition than females^3^ due to their higher competitiveness^47^. However, another study refuted that females were less competitive than males^48^. A study investigating 10-km races showed a significant annual decrease in the male-to-female ratio of finishers, with increasingly more female runners finishing in the sub-hour range^4^. Furthermore, it has been reported that females prefer smaller competitions^49^, which is the case in ultra-marathon running with lower numbers of participants. The sex difference in performance can also be due to the motivating factors to participate in competitions. Between 1975 and 2013, master athletes improved their performance, where the magnitude of improvement was higher in the older age groups leading to gradual closing to younger athletes^50^. It has been reported that the motivation to enter an athletic competition is based on social conditions and predisposition^3^. A study of marathoners aged 20–79 found that sex differences in running speed increased with age, primarily attributed to the lower number of females than males^51^. In marathon running, however, a successful finisher can achieve a similar race performance from 20 to 55 years^52^, which would not be possible in ultra-marathon running. The sex difference in marathon versus ultra-marathon in the 75+ age group may be attributed to the declining number of male marathoners after 2005. Conversely, the number of female marathoners in the Venice Marathon increased from 2003 to 2019, providing a counterpoint^53^.

### Differences in the trend of sex difference by race distance

There was a general trend of decreasing sex difference from the 20–24 age group to the 65–69 age group, except for 5 km and the marathon, which increased after this age. In contrast, the sex difference continuously decreased with increasing age in 10 km, half-marathon, and ultra-marathon races. The male-to-female ratio by age group and distance may explain these trends, as observed in an analysis of races held in Oslo from 2008 to 2018. Female runners comprised a higher percentage of finishers in the 10 km race, but fewer in the half-marathon and marathon, and the male-to-female ratio was lowest in the 10 km and highest in the marathon^54^.

Another explanation could be the age itself. The age of ~ 65–70 years is also important regarding the age-related performance decline^55,56^. In age group athletes, performance declines curvilinear from the age of 35 years until the age of ~ 65–70 years^57^. McClelland and Weyand^16^ recently noted a sex difference of ~ 12% for running distances from 800 m to 10 km, attributed to differences in energy supply and demands. Accordingly, Jobe et al.^58^ observed that males were 9–13% faster than females in all running events of the United States Olympic trials (from 100 m to marathon). Considering the physiological mechanisms underpinning the decrease of sex difference with the increasing race distance, an explanation might be the different taxing of the energy transfer systems in races varying for distance^58^. As the race distance increases, there is a larger contribution of the aerobic processes and a smaller anaerobic mechanism^59^. Thus, the existence of smaller sex differences in aerobic than in anaerobic capacity might relate to the decreased sex difference in increasing distances^60^. This is also in accordance with the latest study by Le Mat et al.^61^, although the race distances analyzed by the authors exceeded the ultra-marathon distances analyzed in this paper (45–260 km) a clear trend of decreasing sex difference with increasing race distance was shown.

### Limitations, strengths, and practical applications

A limitation of the present study was that it analyzed race data from a single country; thus, considering the differences in performance and participation trends among countries, the findings should be generalized with caution to other countries. On the other hand, the strength was the large dataset that allowed drawing safe conclusions about the variation of sex difference by race distance and age group. Another potential limitation is the reliance on descriptive statistics in the result interpretation. The finding has practical applications for scientists and professionals working with long-distance runners to set optimal training goals for female athletes. Specifically, the main goals in training optimization considered should be body composition, sex differences in performance and performance differences throughout the aging process.

## Conclusion

In summary, elderly female ultra-marathoners (age group 75+) show the smallest performance difference from male ultra-marathoners compared to other running distances from 5 km to a marathon. This is probably due to ‘highly selected’ female ultra-marathoners who perform exceptionally well. Future studies might investigate the experience and motivation of elderly female ultra-marathoners.

## Data Availability

For this study, we have included official results and split times from “swiss-running” (www.swiss-running.ch), “runme” (www.runme.ch/de/laufkalender/schweiz), “datasport” (www.datasport.com/de) and “DUV” (https://statistik.d-u-v.org/calendar.php). The datasets used and/or analyzed during the current study are available from the corresponding author on reasonable request.
